# Evaluation of clinical versus non-clinical continuing education in terms of preferences and value for oral healthcare workers

**DOI:** 10.1080/10872981.2022.2125630

**Published:** 2022-09-20

**Authors:** Tony Skapetis, Simran Cheema, Mariam El Mustapha

**Affiliations:** a Sydney Dental School, Faculty of Medicine and Health, University of Sydney, Westmead Campus, Sydney, NSW, Australia; b Division of Oral Health, Western Sydney Local Health District, Sydney, NSW, Australia

**Keywords:** CME, CPD, CE, dental, oral health, healthcare, education, conferences, programs

## Abstract

**Background:**

Continuing professional development (clinical) and continuing education (non-clinical) is fundamental to education and self-improvement of all categories of staff within a large healthcare facility.

**Aim:**

This study sought to examine the attendance preferences and perceived value of clinical and non-clinical oral healthcare workers towards clinical continuing professional development (CPD) and non-clinical, continuing education (CE) activities.

**Methods:**

A retrospective cross-sectional survey design was used capturing 8640 self-reported evaluations collected across 8 successive years and 160 CPD and CE activities in a large dental hospital. Analysis was performed using descriptive statistics including mean scores, independent t-test and cross tabulations using chi-square.

**Results:**

A strongly significant association (p < 0.001) was found between attendee position type (clinical or non-clinical) and attendance preference to either clinical or non-clinical education. Dental assistants, compared to Dentist/Specialist (p < 0.001) found the programs more accurate, relevant, improved their knowledge, would use what was learned and rated the sessions higher overall. Clinical CPD was deemed more relevant (p = 0.025) and improved knowledge (p = 0.01) while non-clinical CE had higher presenter quality (p < 0.001) and overall mean scores (p = 0.015).

**Conclusion:**

There was a preference towards attending clinical CPD over non-clinical CE, by not only clinical, but also non-clinical oral healthcare workers. Non-clinical CE was scored higher by both clinical and non-clinical participants and should therefore be considered for inclusion in CPD education programs with similar settings.

## Introduction

The terms continuing professional development (CPD), continuing medical education (CME) and continuing education (CE) are often used interchangeably. For the purpose of clarity in this research, CME is interpreted as including continuing dental education (CDE) and being part of the wider and often self-directed CPD as required for health professional certification and licensure, while CE refers more broadly to other education.

The state of NSW, Australia, delivers its health services via a series of large health districts. One of these is Western Sydney Local Health District (WSLHD), which provides public health care, including general and specialist dental services to an overall population of almost 1 million people. Since 2011, WSLHD oral health network has been delivering annually between 1 and 2 whole-day CPD and CE activities to over 300 staff as well as attendees from other local health districts, universities, and the private sector. Only the latter were charged a nominal attendance fee to help offset hosting expenses. The purpose of this education is to provide CPD which helps clinicians fulfill their registration requirements and maintain credentialling, as well as providing general CE for non-professional healthcare attendees, namely administrative staff and those not involved with direct patient care. Continuing medical education has been used as a health worker retention strategy [1] and may reduce quality of care complaints [2]. Furthermore, large healthcare organisations and facilities that embed learning and change management into their core business have been shown to improve healthcare outcomes [3]. Continuing dental education has been reported to have organisational benefits such as helping to meet the demands of a flexible and competent workforce, improve inter-professionalism and quality assurance, as well as staff morale and motivation [4].

There are several publications that have evaluated CME activities and conferences at particular points in time and in different countries for different groups of clinicians [5–7]. There does not appear to be any study, however, that has collected data across several successive years from a single, large, oral-healthcare provider organisation.

Given that the dental team in large institutions consists of a broad diversity of staff, both clinical and non-clinical, a presumption could be made that both clinical and non-clinical education sessions should be made available to better support this staff diversity. Such an approach has been advocated by several authors and it has been reported that non-clinical healthcare staff are provided with less training and promotion opportunities [8,9]. This, however, may require the provision of multiple education streams and require additional planning, resources, and costs. For several years, this has been the untested education strategy adopted by WSLHD, in the interest of adult education best practice. The medical and dental literature although rich in advising aspects of clinical education, is more limited when considering guidance for the education of non-clinical staff. Furthermore, non-clinical healthcare staff are provided with less training and promotion opportunities

The aims of this study were to analyse a large sample of oral healthcare staff responses across several years, towards mostly lecture-based CPD and CE, in order to inform on the following questions:

1. What is the attendance preference and self-reported evaluation of whole-day CPD/CE activities among clinical and non-clinical oral healthcare workers?
2. Is clinical CPD and non-clinical CE valued differently among groups of oral healthcare workers?

## Methods

A retrospective cross-sectional study design was employed involving voluntary, anonymous evaluation questionnaires, completed by attendees, in either paper or electronic (Survey Monkey) format collected between 2012 and early 2020, n = 8640. As this was a retrospective study, the sample size was a consequence of the years across which responses had been collected, rather than any power calculations. There were 160 CPD/CE sessions of varying duration (mostly 1 hr), delivered over 13 CPD days which were evaluated across the 8 year period.

Examples of clinical CPD included clinical case presentations, specialty and discipline-specific topics (paedodontics, prosthodontics, special needs, endodontics), infection control, diabetes management, and medical emergencies. Examples of non-clinical CE, included customer service, personal inspiration, leadership, empowerment, cultural competence, communication, mindfulness, and meditation. Participants were mostly local health district employees together with University, private practice clinicians and Australian Defence Force, oral health personnel.

The eight survey instrument questions (Table 1) had been tested during previous CPD/CE activities within Westmead Centre for Oral Health for content validity and responses measured against a 5-point Likert scoring scale (highest to lowest). The instrument testing involved administering the survey to several hundred respondents during previous CPD/CE activities (not included in the current analysis) and refining it by making it anonymous (previously identifiable), simplifying the wording and removing questions that were repetitive. Although the exact wording of the questions was original, it was adapted from previous publications [6,10]. Also included were demographic questions including professional and employment classification, work location together with the choice of session attended. Survey responses were entered into SPSS version 26 with cross-checking of entries by 2 of the authors. Data analysis was performed using descriptive statistics including mean scores, independent t-test and cross tabulations using chi-square. Statistical significance was set at p < 0.05.

**Table 1.** Survey instrument questions (non-demographic).

Q1	The presentation followed the description in the program
Q2	The information presented was relevant
Q3	The audio-visual aids were effective
Q4	There was enough time for questions and answers
Q5	I improved my knowledge as a result of this presentation
Q6	I will be able to use what I have learned today in my future practice
Q7	I would rate the quality of the presenter as:
Q8	Overall, I give the presentation a score of:

## Results

Of the 8640 respondents, 8264 identified their category of employment as presented in Table 2. Overall, clinical staff responses constituted just over 91% of the total responses and the remainder were non-clinical. All eight questions displayed a non-normal data distribution using the Kolmogorov-Smirnov test (p < 0.01) that was heavily skewed towards higher value scores.

**Table 2.** Employment category of participants.

Category of Employment	Percentage
**Clinical**	
Dentist/Specialist	34.4
Dental Assistant	31.7
Oral Health Therapist/Dental Therapist	19.5
Lecturer/Educator	4.7
Nurse	0.5
Hygienist	0.2
Prosthetist	0.3
**Non-clinical**	
Administration/Clerical	4.5
Researcher	0.1
Dental Technician	2.1
Other	2

Of those participants that responded to the question on employment type, the majority were employed by public sector local health districts (85.7%) with smaller percentages of participants identifying with other employment, universities, or private sector. (Table 3)

**Table 3.** Employment workplace of participants.

Place of Employment	Frequency	Valid Percent
Western Sydney Local Health District (LHD)	4576	58.4
South Western Sydney LHD	782	10
Other	710	9
Sydney LHD	390	5
South Eastern Sydney LHD	258	3.3
University	256	3.3
Northern Sydney LHD	229	2.9
Nepean/Blue Mountains LHD	196	2.5
Illawarra/Shoalhaven LHD	145	1.9
Private practice clinician	133	1.7
Western NSW LHD	69	0.9
Monash Health Melbourne	36	0.5
Central Coast LHD	23	0.3
Australian Defence Forces	23	0.3
Totals	7826	100

The overwhelming majority of responses (>90%) were from clinical staff and reported on clinical CPD (81%). Responses relating to evaluations for clinical and non-clinical sessions are presented in Table 4. Of the 160 CPD sessions, 80% were clinical. Cross-tabulations showed a strongly significant association (p < 0.001) between attendee position type (clinical and non-clinical) and attendance to either clinical CPD or non-clinical CE.

**Table 4.** Type of CPD attended relative to staff position.

		Staff Classification	
CPD Type Attended	Clinical	Non-clinical	Total
Clinical CPD	6107	468	6575
Non-clinical CPD (CE)	1427	248	1675
**Totals**	7534	716	8250

Additionally noteworthy, was that 7.1% of feedback regarding clinical CPD sessions came from non-clinical participants who also provided 14.8% of non-clinical session feedback. Clinical staff provided 92.8% and 85.2% of clinical and nonclinical CPD session feedback, respectively.

Dental assistants (DA) mean score results for the eight survey questions (Q’s) when compared to Dentist and Dental Specialists’ responses (using independent t-test, with equal variance not assumed), recorded significance of p < 0.001 for 5 of the 8 questions as per Table 5. DA’s found the program more accurate (Q1), the information more relevant (Q2), improved their knowledge more (Q5), more likely would use what they learnt (Q6) and rated the presentations higher overall (Q8).

**Table 5.** Question responses from dentist/specialist compared to dental assistants.

	Position	N	Mean	Std. Deviation	Std. Error Mean	Significance (2-tailed)^a^
Q1	Dentist/specialist	2787	4.4316	.70526	.01336	.000
	DA	2559	4.5029	.73867	.01460	
Q2	Dentist/specialist	2789	4.3499	.76426	.01447	.000
	DA	2561	4.4561	.78842	.01558	
Q3	Dentist/specialist	2784	4.3297	.81473	.01544	.096
	DA	2551	4.3685	.87708	.01737	
Q4	Dentist/specialist	2754	4.2774	.85787	.01635	.096
	DA	2463	4.3187	.92374	.01861	
Q5	Dentist/specialist	2787	4.2189	.86995	.01648	.001
	DA	2551	4.3034	.91933	.01820	
Q6	Dentist/specialist	2790	4.0864	.95548	.01809	.000
	DA	2547	4.2057	1.01253	.02006	
Q7	Dentist/specialist	2788	4.3992	2.02378	.03833	.535
	DA	2562	4.4251	.82949	.01639	
Q8	Dentist/specialist	2688	4.2969	.80543	.01554	.001
	DA	2321	4.3770	.85630	.01777	

^a^t-test for equity of means with equal variance not assumed.

When comparing survey response mean scores from clinical versus non-clinical CPD (Table 6), with equal variances not assumed, significance was identified in relation to Q1 (p = 0.006), Q2 (p = 0.025), Q5 (p = 0.01), Q7 (p < 0.001) & Q8 (p = 0.015). Based on these results, attendees more strongly agreed that the clinical CPD sessions had more closely followed the program synopsis (Q1), were more relevant (Q2) and had improved knowledge (Q5). Conversely, non-clinical CPD had significantly higher mean scores in terms of presenter quality (Q7) and overall presentation scores (Q8).

**Table 6.** Question responses relative to clinical CPD and non-clinical CE.

	CPD Session Type	N	Mean	Std. Deviation	Std. Error Mean	Significance (2-tailed)^#^
Q1	Clinical	6702	4.4749	.72573	.00886	.006
	Non Clinical (CE)	1723	4.4167	.79589	.01917	
Q2	Clinical	6711	4.4080	.77411	.00945	.025
	Non Clinical (CE)	1724	4.3579	.83807	.02018	
Q3	Clinical	6696	4.3744	.82315	.01006	.152
	Non Clinical (CE)	1720	4.2878	2.47201	.05961	
Q4	Clinical	6561	4.3166	1.01012	.01247	.100
	Non Clinical (CE)	1675	4.2734	.94242	.02303	
Q5	Clinical	6695	4.2714	.88694	.01084	.010
	Non Clinical (CE)	1717	4.1823	1.36043	.03283	
Q6	Clinical	6688	4.1356	1.00559	.01230	.644
	Non Clinical (CE)	1713	4.1483	1.01402	.02450	
Q7	Clinical	6707	4.4096	1.44715	.01767	.000
	Non Clinical (CE)	1727	4.3063	.89900	.02163	
Q8	Clinical	6249	4.3607	1.45147	.01836	.015
	Non Clinical (CE)	1723	4.2919	.89526	.02157	

^#^t-test for equity of means with equal variance not assumed.

When comparing mean score results from the 8 Q’s in terms of clinical versus non-clinical respondents, no significance was recorded. Based on this result, when both clinical and non-clinical attendee responses were combined, Q1 gave the highest mean score value and Q6 the lowest score, although both questions still had high overall mean scores.

## Discussion

Continuing education in Dentistry has been associated with improving confidence, maintenance of competency, improve satisfaction as well as acting as a vehicle for improving career opportunities [4]. More recently, the need for critical, problem thinking and leadership skills as part of a lifelong learning journey have been highlighted for Dentistry [11,12].

There is evidence that CME alone may have a moderate effect in persuading health professionals to follow recommended practices. Furthermore, it is purported that CME alone would likely improve patient health with moderate certainty [13]. Academic health centres are under more pressure and scrutiny to improve clinical and educational outcomes [14]. The same author also describes that there are many impediments towards this greater goal which include workload, culture and organisational challenges. Barriers to continuing dental education have been reported to included time limitations, financial considerations and choice of topics [15,16]. These are all relevant to the large dental hospital setting across which the current study was conducted. Although there were 2 whole day education events planned for each year, only a single CPD/CE day was conducted across four of the eight years.

This study relied on voluntary responses to a questionnaire type survey instrument as this type of methodology have been widely used in health to measure perceptions, attitudes, knowledge and behaviour of clinicians [17]. It has been suggested that poor content may be a leading cause for dissatisfaction with presenters in continuing medical education [6], however in the current study, presenters were rated 3^rd^ highest in terms of mean scores. On the other hand, questions relating to whether presentations improved knowledge (x = 4.25) and the ability to use the knowledge in future practice (x = 4.14), that is, behaviour change, resulted in the lowest mean scores. This, although disappointing, has certain positives attached to these results. Firstly, the results align with other CPD literature which states that medical CPD targets mostly learning rather than higher order behaviour change [18] and this equates to Kirkpatrick’s evaluation model, level 2 (learning), rather than the higher level 3 activities (behaviour change) [19]. Secondly, the fact that lower behaviour change scores were accurately captured by the survey instrument lays testament to its construct validity. It is also noteworthy that the survey question wording; ‘I will be able to use what I have learned today in my future practice’, implies a future change in practice, which is a higher order evaluation result beyond the more easily achieved measures of reaction and learning.

The program content for each CPD/CE day was largely based on several influencing factors all of which regularly affected the final content including any repetition. These included, a previous training needs analysis (conducted in 2008 and 2010 that identifying topic preferences), health district imperatives and strategy at the time (for example infection control audit results and managing aggressive behaviour shortfalls), costs, availability of speakers and resources (space availability) as well as topics put forward from staff and executive. Additionally, there was more emphasis placed by the program organisers in providing CPD rather than CE sessions, given the majority of attendees were from a clinical background. This may in turn bias the results.

Financial barriers resulted in speakers mostly donating their time on an honorary basis as well as some presenters being sponsored by pharma, the dental industry, insurance companies (medical indemnity) or professional associations. The sponsorship was limited to having a speaker available to present on a CPD topic as chosen by the sponsor which was seen to be aligned with the education needs of the public sector. None of the CE sessions were sponsored. Overall, the majority of the CPD sessions were not subject to sponsorship with approximately one-quarter (23%) being sponsor supported.

Time, costs, and distance have been reported as the most important barriers for dentists not attending CPD [20]. These CPD/CE days were not only provided at no cost to the majority of participants who were public sector employees, but additionally, they were paid their normal salaries during participation. This may have biased the survey results, likely in a positive direction.

The survey question around relevancy of the education sessions produced an overall mean score only second to the question concerned with the accuracy of the program description. This may be explained by the fact that the education topics were well aligned with the education needs of participants which in turn was assisted by providing several parallel steams during each education day. In this way, both clinicians and non-clinicians, had greater participatory choice to attend sessions of most interest and alignment to their individual learning needs as is the preference in adult education and lifelong learning strategies [21,22]. Furthermore, another explanation could be the overwhelming majority of clinical attendees which aligned well with the predominance of CPD over CE sessions.

The results demonstrated a strong statistical significance (p < 0.001) between attendee position (job classification) and attendance patterns. Namely, both clinical and non-clinical attendees had a strong preference for attending clinical CPD. This was further supported by the significant differences in the mean scores of 5 of the 8 questions related to clinical versus non-clinical CPD/CE.

A booklet containing the program with speaker generated abstracts and biographies, was distributed among attendees to support each CPD day as would be typical of other health-related CPD conferences. Sponsored sessions were clearly inclusive of logos, however, program booklets did not identify the sessions as either CPD or CE. This may help explain the Q1 scores (p = 0.006) where participants may be more use to reading clinical CPD abstracts compared to non-clinical, as the latter may be more vague and subjective. Similarly, for Q2 scores (p = 0.025), the information provided was perceived more relevant given it was clinical and scientifically based compared to the non-clinical CE which was more abstract in nature. Also, the significance in learning response (Q5, p < 0.01) mean scores, between clinical & non-clinical CPD/CE, is not surprising, given attendees were mostly clinical and would likely build on their existing clinical knowledge more, following clinical CPD.

Conversely to the above findings, non-clinical presenter quality was scored significantly higher (p < 0.001), together with overall presentation scores (p = 0.015) in comparison to clinical. An explanation may be that these non-clinical CE presenters may have brought an element of novelty, be more engaging and less boring and more likely to have used different methods of presenting, for example, used more audience interactivity, which has been shown to be more effective [23].

Proportionately more clinical staff preferred to attend clinically based CPD sessions, however, non-clinical staff also showed a preference for attending clinical sessions despite freedom of session choice and fundamental differences in work duties. This may be explained in part by the fact that there were more clinical CPD sessions offered compared to non-clinical (4:1 ratio). Dental assistants (DA) rated the presentations higher for the majority of questions when compared to dentists and dental specialists. One explanation may be that DA’s knowledge gap was greater compared to other clinical staff and therefore were able to benefit more from the CPD education.

Non-clinical CE sessions had a higher overall presentation score. This may be explained by the fact that these sessions were often more engaging with many focused on improving communication, mindfulness, wellbeing, and other non-technical subjects thus allowing participants to more easily relate to the subject matter. Furthermore, the non-clinical CE may have been viewed as somewhat refreshing and less common.

Limitations of the study include inherent weaknesses associated with the survey instrument such as only having 2 demographic questions (position and workplace), possible errors in lecture allocation as clinical or non-clinical, missing responses to questions, possibility of multiple responses from same attendees, the unknown number of session participants and non-respondents and the non-uniformity of sessions and respondents. Additionally, due to the large sample size, differences in the question absolute scores, showing significance, were relatively small and therefore may be limited in terms of practical relevance. Strengths include the large sample size, instrument validation, cross-checking of data entry into SPSS and the novelty of this under-reported area of study.

Future research may compare ratings from sessions and topics which were mandated by the employer versus other CPD/CE. Additionally, the results relate largely to the pre-COVID-19 pandemic with more traditional face-to-face CPD/CE activities and may differ from the more recent shift towards virtually delivery due to COVID-19 [24–26].

## Conclusion

Clinical CPD was generally attended more by clinical and non-clinical participants compared to non-clinical CE in a large dental hospital setting across an 8 year period. This may help dispel the assumption that non-clinical participants would have a preference for attending non-clinical CE. Despite this, non-clinical CE was scored higher by both clinical and non-clinical participants and should be considered for inclusion in any CPD/CE program within a similar context.

Dental CPD/CE in the form of both clinical and non-clinical content was highly valued by both non-clinical and clinical participants alike. Of the latter participant group, DA’s responded more favourably to most survey evaluation questions.

## Data Availability

Raw data were generated at Westmead Centre for Oral Health. Derived data supporting the findings of this study are available from the corresponding author on request.
